# Preemptive analgesia for hemorrhoidectomy: study protocol for a prospective, randomized, double-blind trial

**DOI:** 10.1186/s13063-022-06107-0

**Published:** 2022-06-27

**Authors:** Ekaterina Kazachenko, Tatiana Garmanova, Alexander Derinov, Daniil Markaryan, Hanjoo Lee, Sabrina Magbulova, Petr Tsarkov

**Affiliations:** 1 Surgical Department, Moscow Research Educational Center of the Lomonosov Moscow State University, Moscow, 101000 Russia; 2 Department: Clinic of Colorectal and Minimally Invasive Surgery, Sechenov First Moscow State Medical University, Moscow, 101000 Russia; 3 Section of Colorectal Surgery, Department of Surgery, New York Medical College, Westchester Medical Center, Taylor Pavilion, Suite D-361, 100 Woods Road, Valhalla, NY 10595 USA

**Keywords:** Analgesics, Anorectal surgery, Hemorrhoidectomy, Pain management, Preemptive analgesia

## Abstract

**Background:**

Hemorrhoidectomy is associated with intense postoperative pain that requires multimodal analgesia. It includes nonsteroidal anti-inflammatory drugs (NSAIDs), acetaminophen, and local anesthetics to reach adequate pain control. There are data in literature preemptive analgesia could decrease postoperative pain after hemorrhoidectomy. The aim of this study is to assess the efficacy of preemptive analgesia with ketoprofen 100 mg 2 h before procedure per os with spinal anesthesia to decrease postoperative pain according to visual analog scale and to reduce the opioids and other analgesics consumption.

**Methods:**

Patients of our clinic who meet the following inclusion criteria are included: hemorrhoids grade III–IV and the planned Milligan-Morgan hemorrhoidectomy. After signing the consent all participants are randomly divided into 2 groups: the first one gets a tablet with 100 mg ketoprofen, the second one gets a tablet containing starch per os 2 h before surgery (72 participants per arm). Patients of both arms receive spinal anesthesia and undergo open hemorrhoidectomy. Following the procedure the primary and secondary outcomes are evaluated: opioid administration intake, the pain at rest and during defecation, duration, and frequency of other analgesics intake, readmission rate, overall quality of life, time from the procedure to returning to work, and the complications rate.

**Discussion:**

Multimodality pain management has been shown to improve pain control and decrease opioid intake in patients after hemorrhoidectomy in several studies. Gabapentin can be considered as an alternative approach to pain control as NSAIDs have limitative adverse effects. Systemic admission of ketorolac with local anesthetics also showed significant efficacy in patients undergoing anorectal surgery. We hope to prove the efficacy of multimodal analgesia including preemptive one for patients undergoing excisional hemorrhoidectomy that will help to hold postoperative pain levels no more than 3–4 points on VAS with minimal consumption of opioid analgesics.

**Trial registration:**

ClinicalTrial.govNCT04361695. Registered on April 24, 2020, version 1.0.

**Supplementary Information:**

The online version contains supplementary material available at 10.1186/s13063-022-06107-0.

## Background

Anorectal diseases are mostly benign and do not affect life expectancy, so currently both patients and clinicians are extremely interested in performing day care surgery. Anorectal surgery itself causes certain discomfort, especially pain, long recovery period, and a decreasing quality of life for several months.

The pain appears as a result of endogenous and exogenous factors affecting on the peripheral nerves endings, and also the pathological central nervous system excitation. Any surgical intervention activates nociceptors with various stimuli including mechanical factors as a result of a prick or cut of the tissue, chemical factors as a result of exposure to inflammatory mediators, and thermal factors as a result of heating or cooling the tissue [1]. Thus, any surgery is always accompanied by a pain syndrome with various pain severities.

In anorectal surgery, almost every patient experiences moderate/severe pain in the postoperative period, 12% of patients have severe pain throughout the recovery period, and control of postoperative pain is still problematic in 5% of cases when a severe pain syndrome continues despite standard pain management. It leads to long hospital admission and to an opioid intake increase [2]. The problem of analgesia is still relevant for patients after anorectal surgery, because the diseases affect more than 50% of the population over 50 years [3]. Excisional hemorrhoidectomy is the most effective method of stage III–IV hemorrhoidal disease treatment. However it is associated with intense postoperative pain which decreases significantly the quality of life in the postoperative period and overall patients’ satisfaction with treatment, increases time spent in hospital, and opioid analgesics consumption [2, 4]. The pain after hemorrhoidectomy (HE) and other anorectal surgery depends on anal sphincter and puborectal muscles spasm, the type of intra- and postoperative anesthesia, wound healing, surgical technique, stool type, and patient’s subjective perception [5, 6]. According to pain management international guidelines, the target level of postoperative pain should be 3–4 or less visual analog score (VAS) points [7].

Preemptive analgesia is aimed to prevent pain after surgery and affects several points of the “pain” cascade [2]. The most common non-narcotic analgesics that block peripheral pain perception include NSAIDs, corticosteroids, and acetylsalicylic acid. Non-narcotic drugs that inhibit central sensitization include ketamine, acetaminophen, and some anticonvulsants (in particular, gabapentin). So multimodal analgesia could theoretically reduce pain to a minimum due to blocking all kinds of pain receptors. However, the preoperative use of even one type of analgesic contributes to pain relief and to the opioid consumption decrease after the intervention [8–11].

One of the most commonly prescribed painkillers is non-steroidal anti-inflammatory drugs (NSAIDs), which have an adequate analgesic effect and even in acute pain can reduce the opioid dose by 18.3% [12]. Regarding ketoprofen as an analgesic drug, none of the published studies describes anorectal surgery. However, ketoprofen is widely used in spinal, orthopedic, general, dental, and children surgery with successful outcomes [13–16]. It also showed significant efficacy as preemptive analgesia compared to the other drugs [17]. Ketoprofen is approved for use as an analgesic for the treatment of mild to severe pain in the postoperative period in total daily doses up to 300 mg; the recommended initial dose is 25 to 100 mg (the dosages are intended for people without acute or decompensated concomitant diseases or non-pregnant women) [18]. Comparing ketoprofen to acetaminophen, the first one showed a significantly better anti-inflammatory effect on the 3rd and 6th day after surgery and lower pain intensity [19].

Therefore adequate postoperative analgesia requires the multimodal approach including opioid analgesics, nonsteroidal anti-inflammatory drugs (NSAIDs), acetaminophen, and local anesthetics. But there is no current standard on preemptive analgesia for adult patients with symptomatic hemorrhoids grade III–IV undergoing Milligan-Morgan hemorrhoidectomy without previous history of anorectal interventions and decompensated somatic diseases, although several studies report some regimens that seem to be effective.

## Methods

### Objective

The aim is to assess the efficacy of preemptive analgesia with Ketoprofen 100 mg 2 h before procedure per os with spinal anesthesia to decrease postoperative pain according to visual analog scale and to reduce the opioids and other analgesics consumption.

### Study design and setting

This is a prospective, randomized, double-blind, unicenter, superiority, parallel-group 2-arm study with 1:1 allocation ratio conducted in the surgical department of Moscow Research Educational Center of the Lomonosov Moscow State University. It is in the recruitment stage. We are anticipating 144 patients of all genders from 18 to 75 years old in total who come to the clinic. Thus, in this case, a uni-center design can assure sufficient patient recruitment.

### Eligibility criteria

Every patient included in the study must meet the following criteria:

- Symptomatic hemorrhoids grade III–IV
- Planned surgery: Milligan-Morgan hemorrhoidectomy

Patients who had contraindication or technical inability to perform subarachnoid anesthesia or other somatic diseases (history of peptic ulcer diseases and gastrointestinal bleeding and other acute or decompensated organ pathology) or refused to participate and pregnant women are not included. The written voluntary informed consent to participate (Additional file 3) is obtained from all eligible patients before randomization.

### Interventions

#### Preoperative preparation

Two hours before the procedure, every patient receives a medication. The research group receives ketoprofen 100 mg per os; the control group receives a placebo.

#### Surgical technique

Under spinal anesthesia, the patient is placed in a modified lithotomy position on the back, with legs spread apart on supports. During anesthesia, the patient is sitting. The needle is inserted between L3 and L4 space or L4 and L5 space. We usually use a bupivacaine solution 5 mg/ml for spinal anesthesia. No adjuvant medicaments are used. The operative field is treated with an antiseptic solution twice and draped. A complex of external and internal hemorrhoids or internal hemorrhoids only is excised with monopolar electrocautery or bipolar electrosurgery device. Hemorrhoid pedicle is tied with an absorbable polyfilament suture, and then the pedicle is crossed by monopolar or bipolar electrocautery. The wound is not sutured. One, two, or three nodes can be removed per procedure depending on the number of the enlarged hemorrhoidal nodes.

#### Postoperative period

After the procedure, all patients receive the standard analgesics scheme. It includes ketoprofen (the average dose, 100 mg per day; the maximum dose, 200 mg per day), paracetamol (at the maximum dose of 1000 mg per day), and local anesthetics with lidocaine before defecation. Patients are usually discharged on the 4–5th day after surgery when their pain level is below 5 points according to VAS. After discharge, a standard analgesics scheme mentioned above is recommended to all patients. If the pain level reaches 5–6 points according to VAS on the 5th day after the procedure, patients continue inpatient treatment and may receive an opioid analgesic.

### Baseline characteristics

The list of the baseline characteristics that we collect to evaluate the population:

- Gender
- Age
- Hemorrhoid stage
- The number of enlarged hemorrhoids
- Hemorrhoid symptoms

### The main outcome measures

The trial is conducted to evaluate the primary outcome: the quantity of the opioid administration intake per day during the first week postoperatively that is necessary to hold pain level no more than 3–4 VAS points in every patient. We use tramadol as an opioid analgesic in a single standard dose of 100 mg for patients with persistent severe pain syndrome in our clinic. The study also assesses the following secondary outcomes: (1) the pain severity before and after defecation according to VAS on the 6, 12, and 24 h after the procedure, then 2 times per day up to 7th postoperative day; (2) duration and (3) frequency of other analgesics intake (systemically and topically) during the first week postoperatively; (4) readmission rate and (5) overall quality of life on the 7th and 30th days; (6) time from the procedure to return to work; and (7) the complications rate (i.e. bleeding, retention of urine, infectious complications) in the early postoperative period (30 days after procedure). The pain severity, duration and frequency of other analgesics intake during the first week postoperatively will be assessed using “Postoperative protocol for pain management” (Additional file 2). The overall quality of life will be assessed with a patient-reported questionnaire Short Form 36 (SF-36). A total score in each of the 8 sections will be calculated and transformed into a 0–100 scale with a score of zero equivalents to maximum disability and a score of 100 equivalents to no disability. Other secondary outcomes will be updated during visits or by phone on the 7 and 30 days after the surgery.

All patients are scheduled to return to the ambulatory clinic on the 7 and 30 days after the surgery. During these visits, postoperative data is collected and digital rectal examination is performed. If a patient fails to follow up, the researcher may contact the patient by all means available (phone, email, or mail) to ascertain whether the patient has had any complications and/or adverse events that were treated at another hospital. If the researcher is unsuccessful in contacting the patient, the patient will be considered as lost to follow-up.

### Participant timeline

For the schedule of enrolment, interventions, and assessments, see Table 1.

**Table 1:**
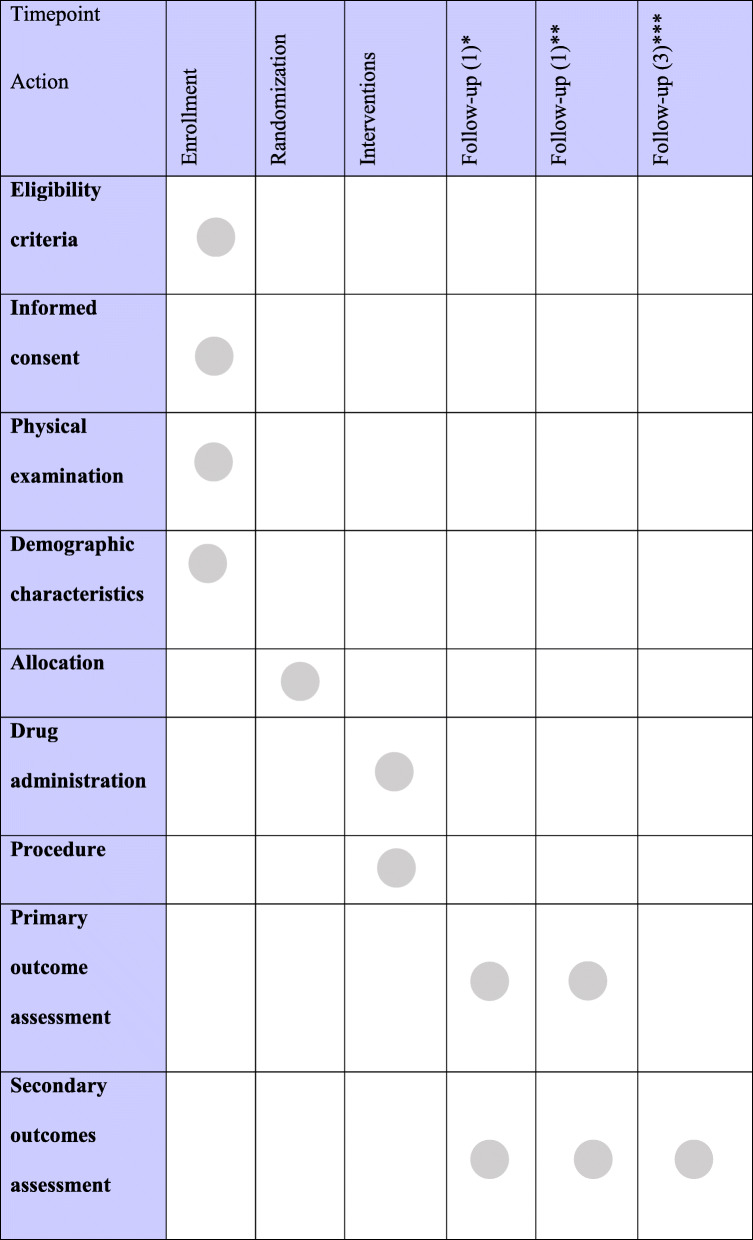
Schedule of enrollment, interventions, and assessments

*Follow-up (1)—the first postoperative day

**Follow-up (2)—the first week after the procedure

***Follow-up (3)—postoperative day 30

### Sample size

Considering that this is a superiority study, the sample size was calculated using a 1-sided Blackwelder test. According to published data, the incidence of opioid intake after hemorrhoidectomy varies from 20 to 30% [7]. The expected incidence of opioid intake after hemorrhoidectomy with preemptive analgesia is not more than 10%. The purpose of this study is to show that the opioids intake in patients with preemptive analgesia is lower than without it. Considering that *a* = 0.05. the statistical power of the study is 80%, the patients are randomized into 2 groups with 1:1 allocation ratio, the noninferiority margin *D* = 5%, and the required sample size is 144 patients (72 patients in each of the 2 groups).

### Recruitment

All patients diagnosed with HD II–III stage will be considered for this study.

### Assignment of interventions

**Fig. 1:**
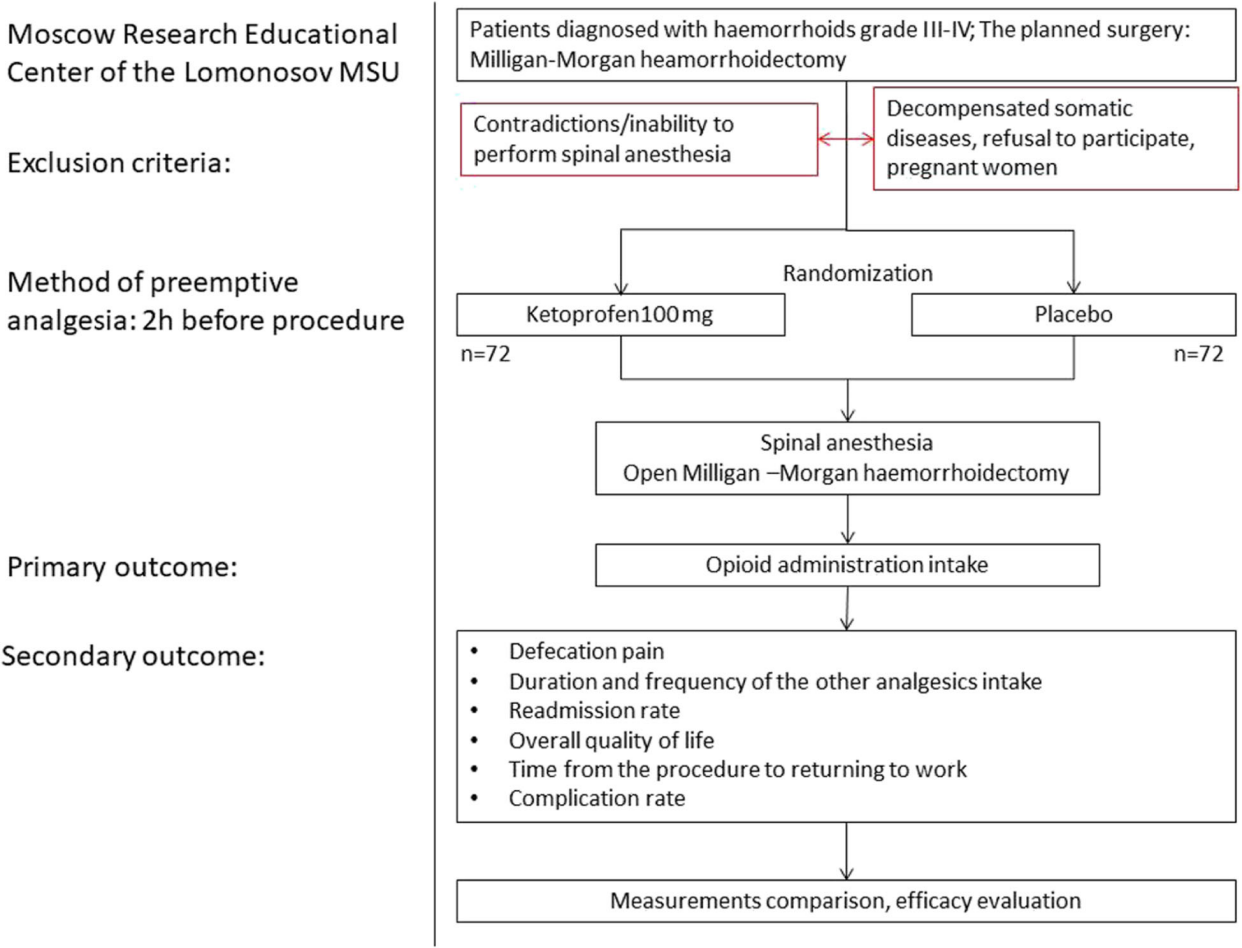
Trial flow diagram

Participants will be randomly assigned to either control or experimental group with a 1:1 allocation ratio using cluster randomization with a computerized random number generator. All subjects will be allocated any interventions. The experimental group receives a tablet with 100 mg ketoprofen, the control one receives a tablet containing starch per os 2 h before surgery (72 participants per arm) (see Fig. 1). The investigator who does not operate generates the allocation sequence, enrolls participants, obtains informed consent, and assigns participants to interventions. The surgeon and the anesthesiology team are blinded.

All relevant data from patient charts except patients’ names will be transferred into an electronic case report form (eCRF). The eCRF should contain results of all the screening procedures, including patient history and demographics, imaging studies, filled-out questionnaires, operation notes, and postoperative rounds during a patient stay in the surgical ward.

### Data collection, management, and analysis

All data will be collected prospectively using eCRFs designed for this trial. The reasons for withdrawal will be documented. The investigator will attempt to contact each participant at least 3 times during each follow-up window before declaring them lost for observation. The study exit form will be recorded in the eCRF. All prior data will be analyzed within the research.

All patients will receive clarifications of all the study procedures and will be able to discuss them with the primary investigator. All patient data will be handled according to the principles of doctor–patient confidentiality; the subjects will be anonymized and analyzed with individual identifier numbers transcribed into eCRF.

Data analysis will be performed by one of the researches who is blind to the randomization by using Statistical Product and Service Solutions (SPSS) Statistics for Windows, version 20.0 (IBM Corp., Armonk, NY, USA). Continuous variables will be presented as mean and standard deviation, and categorical variables will be presented as frequency or percentage.

### Statistics

The ITT analysis included all randomized patients treated for at least 1 week, who were evaluated for efficacy at baseline and at least once at subsequent evaluation points during the study. The PP-analysis will exclude from the full analysis all the non-compliant patients, patients who received incomplete treatment, and patients who refused further treatment after randomization.

Quantitative variables are described as means with standard deviations, medians, range, or interquartile range as appropriate. Categorical variables are described in absolute numbers and percentages. The statistical analysis of the quantitative variables, with independent groups, is performed with the parametric Student’s *t* test, providing that its conditions for application are met. Otherwise, the non-parametric Mann-Whitney *U* test is used. Statistical analysis for categorical variables is performed using the Pearson *χ*^2^ test or the Fisher exact test. Specifically, the above methods are used to compare the two groups in terms of baseline characteristics in order to assess whether the randomization has been effective. Ninety-five percent confidence intervals for the ratio will be reported. In all cases, the level of significance will be the usual 5% (*α* = 0.05).

This study has a low risk of selection bias due to the use of a computer random number generator. Central allocation and the use of sequentially numbered drug containers of identical appearance allow to achieve a low risk of deviations. Performance bias may be considered as having an average risk due to blinding only participants of the study, the personnel is not blinding. Blinding of outcome assessment is ensured, so detection bias is unlikely. Attrition bias could probably appear because of the difficulty of collecting data from patients outside the hospital or the refusal of future treatment and visiting the doctor during follow-up.

### Data monitoring

There is no data monitoring committee designated for this trial. Any adverse and serious adverse events will be immediately reported to the principal investigator and the primary sponsor. Participants’ names and collected data are subject to medical confidentiality. In cases of withdrawal, collection of data will cease but not be erased. A logistics database with a patient’s complete ID will be used and kept within a separate password-protected system from the results database with all study information. At the end of the trial, the original peri-operative package and database will be archived by the principal investigator, who is responsible for providing data to the trial investigators.

### Ethical approval

This study is conducted in accordance to the principles of the Declaration of Helsinki (Fortaleza, 2013). The study protocol is approved by the Local Ethics committee of Sechenov University (see Additional file 1).

### Protocol amendments

Any protocol amendments that may influence the conduct of the study will be communicated to the local ethics committee and study director and will be uploaded to clinical trials.

### Consent or assent

A member of the research team will obtain the consent form. All participants will be able to address their questions about the study to one of the members of the research team.

### Confidentiality

All patient data will be secured at the study site. No one apart from the members of the research team will have access to any patient data, including anonymized eCRFs with a coded ID, as well as filled out questionnaires.

### Declaration of interests

The authors declare they have no competing interests.

### Access to data

No one apart from the members of the research team will have access to the final trial dataset.

### Ancillary and post-trial care

If a study participant receives a complication or suffers in any way, he will be provided with a full recovery in our clinical center free of charge.

### Dissemination policy

Trial results will be e-mailed to all participants of the trial. Trial results will be disseminated to healthcare professionals via publication in a peer-reviewed scientific journal and by mass media, as well as conference papers to inform the public and stakeholders, and will be uploaded to the primary registry. We have no intention of granting public access to the full protocol, participant-level dataset, and statistical code.

## Discussion

Multimodality pain management for anorectal surgery has been shown to improve pain control and decrease opioid requirements in several studies. Preoperative oral acetaminophen and gabapentin admission followed by intravenous ketamine in the early postoperative period resulted to significantly less pain level postoperatively and decreased narcotics intake [2]. Place RJ [20] showed a significant decrease of postoperative analgesics requirement in addition to reducing voiding problems by systemic admission of ketorolac with local anesthetics in patients undergoing anorectal surgery.

Several studies showed that ketoprofen significantly reduced opioid consumption (by 33%) and improved postoperative analgesia after spinal and abdominal surgery [13–16]. Patients with acute pain who have undergone dental surgery get a meaningful pain relief, a faster onset of effect, the highest peak effect, and the longest duration of action using ketoprofen than other NSAIDs and analgesics [18, 21]. In all mentioned above studies, adverse effects related to ketoprofen were minor and infrequent.

We hope to prove the efficacy of multimodal analgesia including preemptive one for patients undergoing excisional hemorrhoidectomy that will help to hold postoperative pain levels no more than 3-4 points on VAS with minimal consumption of opioid analgesics.

## Trial status

Registered at clinicaltrial.gov number ID NCT04361695, date of registration: April 24, 2020, version 1.0. Recruitment began in April 2020 and is expected to be completed by the end of December 2021.

## Supplementary Information

**Additional file 1.** SPIRIT 2013 Checklist: Recommended items to address in a clinical trial protocol and related documents.

**Additional file 2.** Postoperative protocol for pain management.

**Additional file 3.** Written form of informed consent of the patient.

## Data Availability

Not applicable.

## Abbreviations

VAS: Visual analog score
NSAIDs: Nonsteroidal anti-inflammatory drugs
eCRF: Electronic case report form
