# Supplementary material for: Preemptive analgesia for hemorrhoidectomy: study protocol for a prospective, randomized, double-blind trial
Source: Trials. 2022 Jun 27;23:536. doi: 10.1186/s13063-022-06107-0 (PMC9235219; doi:10.1186/s13063-022-06107-0)
Supplement: Supplementary file 2 — Additional file 2. Postoperative protocol for pain management. [file 13063_2022_6107_MOESM2_ESM.docx]

**Postoperative protocol for pain management**

1. Postoperative pain level according to Visual Analogue Scale ( 0 – no pain, 5 - moderate pain, 10 – unbearable pain)

| Time after surgery | 6 hours | 12 hours | 1 day | | 2 days | | 3 days | | 4 days | | 5 days | | 6 days | | 7 days | | |  |
| --- | --- | --- | --- | --- | --- | --- | --- | --- | --- | --- | --- | --- | --- | --- | --- | --- | --- | --- |
| At rest |  |  |  |  |  |  |  |  |  |  |  |  |  |  | |  |  | |
| During defecation |  |  |  |  |  |  |  |  |  |  |  |  |  |  | |  |  | |

1. Analgesics intake per day

| Day after surgery | 1 | 2 | 3 | 4 | 5 | 6 | 7 |
| --- | --- | --- | --- | --- | --- | --- | --- |
| Analgesics |  |  |  |  |  |  |  |
