# Supplementary material for: Preemptive analgesia for hemorrhoidectomy: study protocol for a prospective, randomized, double-blind trial
Source: Trials. 2022 Jun 27;23:536. doi: 10.1186/s13063-022-06107-0 (PMC9235219; doi:10.1186/s13063-022-06107-0)
Supplement: Supplementary file 3 — Additional file 3. Written form of informed consent of the patient. [file 13063_2022_6107_MOESM3_ESM.docx]

**WRITTEN FORM OF INFORMED CONSENT OF THE PATIENT**

I voluntarily agree to participate in this clinical trial: " Preemptive Analgesia for Hemorrhoidectomy: study protocol for a prospective, randomized, double-blind trial", which is conducted by Ekaterina Kazachenko, Tatiana Garmanova, Alexander Derinov, Daniil Markaryan, Hanjoo Lee, Sabrina Magbulova, Petr Tsarkov on the basis of the Surgical department, Moscow Research Educational Center of the Lomonosov Moscow State University, Moscow, Russia.

I have read carefully(a) this document and understood its contents. I have been provided with detailed explanations regarding the information contained in this document. I was given the opportunity to ask questions about this research, and I got(a) they have comprehensive answers. I was also told who to contact if I have any additional questions. All information about the study, including all explanations and answers to questions, was presented in a language that was understandable to me. I understand that I can refuse to participate in this clinical trial or withdraw from the study at any time without specifying a specific reason, and that my decision will not affect the quality of medical care provided to me.

I authorize, if necessary, to inform my attending (district) doctor that I am participating in this clinical trial. I was informed that the data collected during this study will be documented on separate forms that will contain the patient's identification number, my age and my gender. I agree that my medical data collected during this study can be registered and transferred to the responsible authorities in encrypted (pseudonymous) form in accordance with applicable regulations within the framework of notification and documentation obligations. My consent to the transfer of data cannot be revoked and is a prerequisite for participation in the study.

I also agree that all data concerning my identity and registered in the framework of a clinical trial can be transferred to the representatives of the Researcher, the competent authority, as well as in encrypted form to the Researcher or other research centers for scientific assessments.

I also agree that all data concerning my identity registered in the framework of this study may be transferred to the public health authorities of other countries for monitoring purposes.

I was assured that confidentiality will be respected with respect to all personal data, and they will not be publicly available, since all persons who have access to this data are required to keep professional secrets.

I participate in this study voluntarily and agree to have biological materials (for example, samples of tissue, blood and urine) taken from me and used exclusively for the purposes of this study.

By signing and dating this document, I do not waive my legal rights, which belong to me regardless of whether I am a participant in a clinical trial or not. By signing this document, I also undertake to comply with all the prescriptions, instructions and recommendations of the research doctor, to inform him of all the necessary information about the change in my state of health, the appearance of new complaints and symptoms, about seeking medical help, about taking any medications that I have not used before.

I hereby give my consent to the representatives of the Researcher, representatives of regulatory authorities and members of independent ethics committees to obtain access to my data, medical data and analysis results in order to control the clinical trial and the quality of related documentation.

| **Patient's signature:** |  | ______________________________________ |
| --- | --- | --- |
| **Date (dd/mm/yyyy):** |  | ______________________________________ |
| **Last name, first name, patronymic of the patient:** |  | ______________________________________  ______________________________________ |

I, the undersigned, confirm that the patient (his legal representative) who signed this document has been provided with a detailed explanation of all aspects of the study, and that the patient (his legal representative) understands what this study is, as well as the risks and benefits associated with his participation in this clinical trial.

| **Signature of the medical researcher:** |  | _____________________________________ |
| --- | --- | --- |
| **Date (dd/mm/yyyy):** |  | _____________________________________ |
| **Surname, first name, patronymic of the research doctor:** |  | _____________________________________  _____________________________________ |
